# Supplementary material for: Susceptibility Loci for Type 2 Diabetes in the Ethnically Endogamous Indian Sindhi Population: A Pooled Blood Genome-Wide Association Study
Source: Genes (Basel). 2022 Jul 22;13(8):1298. doi: 10.3390/genes13081298 (PMC9331904; doi:10.3390/genes13081298)
Supplement: Supplementary file 1 [file genes-13-01298-s001.zip › genes-1820984-supplementary.pdf]

Supplementary Materials

Contents

Table S1. Variants significantly associated with type 2 diabetes in the pooled whole blood genome-wide association study.......... 2

Table S2. Nearest genes for the significantly associated variants in the pooled whole blood genome-wide association study.......... 6

Table S3: Literature support for significantly associated T2D genes not listed in T2DKP, DisGeNET and Harmonizome databases 11

References: .... **Error! Bookmark not defined.**

Table S1. Variants significantly associated with type 2 diabetes in the pooled whole blood genome-wide association study

No	SNP Name	Chr	Position	Strand	Alleles	GenTrain.Score	MAF	Coef	P
1	rs1001179	11	34438684	+	[T/C]	0.8970338	17.21757	-29.4149	$9.06X10^{-25}$
2	rs480948	11	96320526	+	[A/G]	0.8483506	49.81387	-38.6569	$2.36X10^{-24}$
3	rs360745	3	12984399	-	[T/C]	0.8165381	33.89273	-26.3035	$2.98X10^{-24}$
4	rs7711236	5	3675819	+	[T/G]	0.8177567	39.32941	-26.1924	$3.87X10^{-24}$
5	rs73219073	21	41841409	-	[T/C]	0.882019	3.409773	-38.2267	$4.62X10^{-24}$
6	rs1653889	7	32347041	+	[A/G]	0.8254703	35.46087	-11.1715	$5.63X10^{-24}$
7	rs17313232	15	69607474	+	[T/C]	0.7855741	39.92257	24.69249	$8.63X10^{-24}$
8	rs4987358	1	169696410	+	[T/G]	0.8921242	20.15748	-91.8405	$9.96X10^{-24}$
9	rs6747727	2	76308348	+	[A/C]	0.8424768	13.84089	-17.0683	$1.03X10^{-23}$
10	rs2164624	10	104253687	-	[T/C]	0.8447312	21.66138	-32.2014	$1.11X10^{-23}$
11	rs8017825	14	89008585	+	[T/C]	0.7777103	24.79254	-24.1074	$1.23X10^{-23}$
12	rs931574	3	123380118	+	[T/C]	0.8534294	22.01077	-14.0805	$1.56X10^{-23}$
13	rs11650852	17	52016092	-	[A/C]	0.8562698	12.15916	-46.7347	$1.65X10^{-23}$
14	rs12313841	12	56462834	-	[A/G]	0.858853	40.40862	-70.4031	$1.96X10^{-23}$
15	rs2967605	19	8404854	-	[A/G]	0.8471626	26.68071	-25.7421	$2.07X10^{-23}$
16	rs385501	5	151190083	-	[A/G]	0.8733098	38.3538	-22.2081	$2.74X10^{-23}$
17	rs4573120	6	30800170	-	[A/C]	0.8715596	36.22778	22.60842	$2.75X10^{-23}$
18	rs951631	10	123526359	-	[T/C]	0.8096277	25.4816	-11.5495	$3.03X10^{-23}$
19	rs9789875	21	38590077	+	[T/C]	0.8659714	44.07991	-23.0001	$3.21X10^{-23}$
20	rs962876	18	44547229	+	[T/C]	0.8289507	48.66284	-31.0841	$3.42X10^{-23}$
21	rs77610104	9	124001717	+	[A/G]	0.7320683	15.35481	-32.6937	$5.29X10^{-23}$
22	rs11058150	12	125357986	-	[A/G]	0.7797861	49.01976	51.27038	$5.82X10^{-23}$
23	rs6790377	3	87502923	-	[T/C]	0.8631262	34.56074	-48.4739	$5.95X10^{-23}$
24	rs9815975	3	159006372	-	[T/C]	0.8713089	14.50041	-81.9027	$5.98X10^{-23}$
25	rs4799327	18	32095029	-	[T/C]	0.8657283	18.55402	-23.6674	$6.38X10^{-23}$
26	rs6036804	20	24452435	+	[A/C]	0.8102413	44.13801	18.83336	$6.77X10^{-23}$
27	rs45615138	16	88703906	-	[T/C]	0.8789126	21.36926	-17.0145	$6.87X10^{-23}$
28	rs2600062	3	2005734	-	[T/G]	0.8356243	39.53194	-21.026	$7.02X10^{-23}$
29	rs10937705	4	6181457	+	[T/G]	0.8670157	24.28235	-58.0672	$7.05X10^{-23}$
30	rs4570167	8	25911808	-	[A/G]	0.8218157	49.27151	549.5858	$7.92X10^{-23}$
31	rs2024578	20	54668835	+	[A/G]	0.7824118	32.3449	-67.1583	$9.92X10^{-23}$
32	rs11050349	12	29669447	+	[A/G]	0.8275709	13.08297	-42.1601	$1.03X10^{-22}$
33	rs4717992	7	64121391	+	[A/G]	0.8377055	37.1859	48.79708	$1.31X10^{-22}$
34	rs4503064	8	10721882	-	[T/C]	0.8761758	47.26778	-67.954	$1.39X10^{-22}$
35	rs683466	9	108180599	+	[T/C]	0.906221	13.45124	-36.0507	$1.53X10^{-22}$

36	rs4855447	3	69754177	-	[T/C]	0.8219197	18.54915	-51.2062	$1.54X10^{-22}$
37	rs893746	2	188051647	+	[A/G]	0.8691419	29.0011	-30.2385	$1.59X10^{-22}$
38	rs4269571	8	116989628	+	[T/G]	0.8937545	46.48461	91.95231	$1.83X10^{-22}$
39	rs62308683	4	88502649	+	[A/G]	0.8246087	7.538659	-33.5284	$1.95X10^{-22}$
40	rs7112956	11	92850499	+	[T/C]	0.8416215	16.26344	-101.689	$2.05X10^{-22}$
41	rs76797216	10	1916047	+	[T/C]	0.774822	7.379474	-104.454	$2.13X10^{-22}$
42	rs34926045	15	68436293	+	[T/G]	0.8450588	42.76964	244.5754	$2.40X10^{-22}$
43	rs2971760	7	131729698	+	[T/C]	0.7530587	33.79646	-220.482	$2.47X10^{-22}$
44	exm2260036	1	231190340	+	[A/G]	0.829042	32.57232	101.1478	$2.57X10^{-22}$
45	rs7027911	9	78543197	+	[A/G]	0.8884114	45.33023	28.82888	$2.59X10^{-22}$
46	rs2061771	19	53407265	-	[T/C]	0.7935601	20.86904	-36.8229	$2.98X10^{-22}$
47	rs1339374	9	35660993	+	[A/G]	0.6359456	17.12966	-28.7299	$3.04X10^{-22}$
48	rs8009579	14	58246841	+	[A/G]	0.8176129	35.56537	188.5193	$3.28X10^{-22}$
49	rs72711389	4	177725611	+	[T/C]	0.8987808	20.36412	-162.15	$3.60X10^{-22}$
50	rs2028964	2	30707491	+	[T/G]	0.7734689	17.57986	-209.361	$3.82X10^{-22}$
51	rs61883849	11	43848354	-	[A/G]	0.9064258	10.65239	-76.0895	$3.91X10^{-22}$
52	rs4738067	8	70056862	-	[T/C]	0.7775877	21.63426	-26.7691	$3.99X10^{-22}$
53	rs73379947	18	7910215	-	[T/C]	0.81701	26.21078	-12.3763	$4.47X10^{-22}$
54	rs719530	7	110250908	+	[T/C]	0.8798997	47.7375	-150.917	$4.69X10^{-22}$
55	rs4955988	3	54625030	-	[T/C]	0.8663906	33.43187	-17.5801	$5.19X10^{-22}$
56	rs78471707	13	86561101	+	[T/G]	0.8954863	12.10208	-24.5885	$5.89X10^{-22}$
57	rs2596906	3	12893085	-	[T/C]	0.82052	44.9136	-48.3849	$6.08X10^{-22}$
58	rs9309473	2	73516855	-	[T/C]	0.8871201	18.20458	38.73006	$6.67X10^{-22}$
59	rs10906653	10	14354208	-	[A/G]	0.8414942	27.89548	54.18027	$6.90X10^{-22}$
60	rs58500584	2	234310742	+	[T/C]	0.8035802	3.903426	-151.193	$7.28X10^{-22}$
61	rs10797649	1	146040040	+	[A/C]	0.740202	48.08431	43.08086	$8.92X10^{-22}$
62	rs12271908	11	113239114	-	[A/G]	0.7609079	22.91464	-24.5801	$9.09X10^{-22}$
63	rs6944414	7	71687637	+	[A/G]	0.81654	42.21265	88.75879	$1.09X10^{-21}$
64	rs67193795	2	148976014	-	[T/C]	0.8431661	32.01818	-12.3323	$1.18X10^{-21}$
65	rs61917776	11	125330325	-	[T/C]	0.7667558	9.920161	-87.5879	$1.23X10^{-21}$
66	rs78715995	9	136222575	+	[T/C]	0.840552	5.719052	-74.061	$1.36X10^{-21}$
67	rs75412658	6	21909772	+	[T/G]	0.7984778	42.14743	-43.8584	$1.57X10^{-21}$
68	rs76326187	6	150597358	+	[A/G]	0.8648639	4.46549	-156.89	$1.70X10^{-21}$
69	rs11877530	18	62348702	+	[A/G]	0.8124005	13.80979	-35.4839	$1.74X10^{-21}$
70	rs73339865	5	177957706	-	[T/C]	0.7491468	8.071928	-227.827	$1.77X10^{-21}$
71	rs742460	16	1355536	-	[A/G]	0.8018193	48.33107	205.2113	$1.88X10^{-21}$
72	rs6887695	5	159395637	+	[G/C]	0.9126133	32.12195	749.9427	$1.88X10^{-21}$
73	rs11130310	3	52640651	+	[T/C]	0.8337012	19.30231	-17.6904	$1.89X10^{-21}$
74	rs80225482	6	42040899	-	[T/C]	0.8723255	23.84027	-51.991	$2.11X10^{-21}$
75	rs4920461	1	18206787	-	[T/C]	0.7935094	17.57062	-134.739	$2.34X10^{-21}$
76	rs2153240	9	35648011	-	[T/C]	0.8501979	36.82969	-183.917	$2.81X10^{-21}$

77	rs9905906	17	7782430	-	[A/G]	0.8477524	17.2694	-34.5557	$3.01X10^{-21}$
78	rs10063787	5	67686376	+	[T/C]	0.8353079	11.12797	-110.676	$3.25X10^{-21}$
79	rs4712460	6	20181954	-	[T/C]	0.8685156	30.17947	-262.743	$3.62X10^{-21}$
80	rs12434181	14	23650652	+	[A/G]	0.8730969	6.266216	-108.199	$3.84X10^{-21}$
81	rs73738318	6	45974495	+	[A/G]	0.7646946	21.8515	-47.8392	$5.08X10^{-21}$
82	rs9535171	13	49243505	-	[T/G]	0.7773587	9.806497	-3509.86	$5.20X10^{-21}$
83	rs3132718	6	29868800	+	[T/C]	0.8702217	44.05073	-296.06	$5.65X10^{-21}$
84	rs61739896	3	52364681	+	[T/C]	0.8924895	2.235508	-294.204	$6.77X10^{-21}$
85	rs4731262	7	125765491	+	[T/C]	0.8951094	27.70236	-37.021	$6.93X10^{-21}$
86	rs28476533	17	77249762	+	[A/G]	0.7989789	21.83827	188.9009	$7.95X10^{-21}$
87	rs12129573	1	73302683	-	[T/G]	0.8139279	29.08553	-17.9015	$9.26X10^{-21}$
88	rs8080678	17	80570203	-	[A/C]	0.8754666	40.35648	463.1458	$9.89X10^{-21}$
89	rs4458115	18	7919379	-	[T/C]	0.859909	19.90434	-17.6194	$1.05X10^{-20}$
90	rs4351004	4	24674985	+	[A/G]	0.8774751	22.7201	-25.6203	$1.15X10^{-20}$
91	rs667725	7	126284347	-	[T/C]	0.8393078	49.04462	-921.687	$1.21X10^{-20}$
92	rs682585	1	182590375	+	[T/C]	0.7813015	46.60204	24.59596	$1.23X10^{-20}$
93	rs2997971	1	237438808	-	[T/C]	0.8730111	10.93767	-22.9386	$1.32X10^{-20}$
94	rs6478453	9	120033303	-	[A/G]	0.8762214	43.42913	53.65811	$1.64X10^{-20}$
95	rs6968910	7	43317192	-	[T/C]	0.8904186	36.75257	-142.741	$1.69X10^{-20}$
96	rs32323	5	12132289	+	[A/G]	0.8210714	36.08853	606.0312	$2.06X10^{-20}$
97	rs13072556	3	160683239	-	[T/G]	0.875758	30.03108	-68.5587	$2.48X10^{-20}$
98	rs1932458	9	18917253	-	[A/G]	0.9176666	45.95931	149.9216	$2.92X10^{-20}$
99	rs3771003	2	205769119	-	[T/G]	0.8632571	37.93846	-100.961	$3.14X10^{-20}$
100	rs920590	8	19793650	+	[T/C]	0.8792908	46.27138	-64.2323	$3.56X10^{-20}$
101	rs13224784	7	128515509	+	[A/G]	0.6926138	17.1631	-50.434	$3.77X10^{-20}$
102	rs9999241	4	7173266	+	[A/G]	0.777869	48.7408	73.72042	$4.63X10^{-20}$
103	rs582937	7	18298727	-	[T/C]	0.8601887	24.35788	-357.772	$1.21X10^{-19}$
104	rs13231717	7	125408035	-	[A/G]	0.8925757	25.1429	-317.137	$1.39X10^{-19}$
105	rs2428918	7	78016414	-	[A/C]	0.8816816	32.9031	74.0138	$1.49X10^{-19}$
106	rs11150737	17	80667490	+	[A/G]	0.7641606	26.14956	1313.788	$1.57X10^{-19}$
107	rs77708863	5	137279499	+	[A/G]	0.8220779	6.894579	-173.741	$1.80X10^{-19}$
108	rs76734228	18	4045192	-	[T/C]	0.8337161	2.407508	-48.2794	$4.29X10^{-19}$
109	rs9297310	8	101736002	+	[T/C]	0.770798	39.34267	351.5545	$4.55X10^{-19}$
110	rs2834623	21	34748996	-	[T/C]	0.8197958	12.09576	-24.5641	$4.71X10^{-19}$
111	rs590616	11	100977744	-	[T/G]	0.9082958	45.65304	-33.947	$4.93X10^{-19}$
112	rs1344542	12	109644349	-	[T/C]	0.8523847	47.47783	20.89444	$4.98X10^{-19}$
113	rs904091	12	83556889	-	[T/C]	0.8919958	34.91664	-15.5754	$5.40X10^{-19}$
114	rs77986901	2	129508850	-	[T/C]	0.8533074	9.993334	264.5331	$5.71X10^{-19}$
115	rs1280414	15	57456550	-	[A/G]	0.868367	8.139166	-1210.25	$6.82X10^{-19}$
116	rs77759376	11	71987494	+	[A/G]	0.8434885	6.354303	-100.995	$7.11X10^{-19}$
117	rs959193	5	178202046	-	[A/C]	0.8732058	12.62213	-51.8175	$7.29X10^{-19}$

118	rs12778455	10	31977821	-	[T/G]	0.7999414	6.837236	-50.2383	$1.20X10^{-18}$
119	rs4945848	6	109930602	+	[T/G]	0.8379158	49.86485	-49.4626	$1.73X10^{-18}$
120	rs61406133	2	239513918	+	[T/G]	0.8139279	5.124663	-446.585	$1.78X10^{-18}$
121	rs60603263	3	161686345	-	[T/C]	0.7927033	13.20774	-82.9058	$1.94X10^{-18}$
122	rs13278603	8	88403614	-	[A/C]	0.8501803	13.41563	-22.8591	$1.97X10^{-18}$
123	rs6844238	4	119146548	-	[T/C]	0.8390408	40.2441	-2.4E+14	$2.39X10^{-18}$
124	rs10922613	1	89356533	-	[A/G]	0.6486367	14.11883	-51.2404	$2.49X10^{-18}$
125	rs9737503	11	336744	+	[T/C]	0.7838537	18.43344	-987.834	$3.73X10^{-18}$
126	rs4437352	5	77116920	+	[T/C]	0.8223203	33.2396	-129.077	$4.24X10^{-18}$
127	rs114471390	2	129576909	-	[A/G]	0.88291	2.935851	-235.352	$1.19X10^{-17}$
128	rs17657660	19	41133615	+	[T/C]	0.8481056	27.76136	-251.406	$2.54X10^{-17}$
129	rs6710692	2	73593191	+	[A/G]	0.8509212	17.85053	-288.703	$2.55X10^{-17}$
130	rs651862	20	19518271	+	[T/C]	0.8703439	39.74953	-19.3452	$3.46X10^{-17}$
131	rs6791903	3	22226594	-	[A/G]	0.7931978	34.75594	-67.2324	$4.99X10^{-16}$
132	rs943121	10	6142950	+	[A/C]	0.7959612	12.21664	-2.9E+14	$2.03X10^{-15}$
133	rs12721515	1	109917523	-	[T/C]	0.8740867	2.465549	-2E+14	$1.47X10^{-14}$
134	rs115128033	7	57372353	-	[T/C]	0.519083	2.32872	-0.40719	$4.38X10^{-13}$
135	rs4661310	1	14993953	+	[T/C]	0.7808229	37.0893	5.68E+14	$1.06X10^{-12}$
136	rs148373909	16	3657256	-	[A/G]	0.7902021	1.574911	-0.71942	$6.54X10^{-10}$
137	rs786201057.1	17	7675995	-	[T/C]	0.585574	1.432765	-0.85771	$1.33X10^{-09}$
138	rs118050771	14	99638359	-	[T/C]	0.631498	1.014242	-1.9085	$1.35X10^{-09}$
139	rs76866981	22	48944900	-	[A/G]	0.6880267	1.180325	-4.1719	$1.36X10^{-09}$
140	rs77194116	8	104355142	+	[T/C]	0.6643735	1.534635	-1.18661	$1.55X10^{-09}$

Table S2. Nearest genes for the significantly associated variants in the pooled whole blood genome-wide association study

No	SNP Name	Nearest Gene				
		Symbol	Entrez ID	HGNC ID	Gene name	Distance (bp)
1	rs1001179	CAT	847	1516	Catalase	241
2	rs480948	MAML2	84441	16259	Mastermind like transcriptional coactivator 2	Overlapping
3	rs360745	IQSEC1	9922	29112	IQ motif and Sec7 domain arfgef 1	Overlapping
4	rs7711236	AC025773.1				336889
5	rs73219073	PRDM15	63977	13999	PR/SET domain 15	Overlapping
6	rs1653889	AC018637.1				80860
7	rs17313232	DRAIC	145837	27082	Downregulated RNA in cancer, inhibitor of cell invasion and migration	Overlapping
8	rs4987358	C1orf112	145837	27082	Downregulated RNA in cancer, inhibitor of cell invasion and migration	Overlapping
9	rs6747727	RN7SKP203	106479185	45927	RN7SK pseudogene 203	136731
10	rs2164624	GSTO1	9446	13312	Glutathione S-transferase omega 1	Overlapping
11	rs8017825	AL137785.1				4801
12	rs931574	ADCY5	111	236	Adenylate cyclase 5	Overlapping
13	rs11650852	CA10	56934	1369	Carbonic anhydrase 10	Overlapping
14	rs12313841	MIP	4284	7103	Major intrinsic protein of lens fiber	Overlapping
15	rs2967605	MARCH2	51257	28038	Membrane associated ring-CH-type finger 2	8416
16	rs385501	CCDC69	26112	24487	Coiled-coil domain containing 69	Overlapping
17	rs4573120	LINC00243	401247	30956	Long intergenic non-protein coding RNA 243	Overlapping
18	rs951631	AL357127.1				47868
19	rs9789875	ERG	2078	3446	ETS transcription factor ERG	Overlapping
20	rs962876	AC120049.1				129698
21	rs77610104	LHX2	9355	6594	LIM homeobox 2	Overlapping
22	rs11058150	TMEM132B	114795	29397	Transmembrane protein 132B	Overlapping
23	rs6790377	APOOP2	100129005	48740	Apolipoprotein O pseudogene 2	91915
24	rs9815975	IQCJ-SCHIP1	100505385	38842	IQCJ-SCHIP1 readthrough	Overlapping
25	rs4799327	AC011825.3				Overlapping

26	rs6036804	SYNDIG1	79953	15885	Synapse differentiation inducing 1	16764
27	rs45615138	RNF166	115992	28856	Ring finger protein 166	Overlapping
28	rs2600062	RN7SKP144	106479159	45868	RN7SK pseudogene 144	83199
29	rs10937705	C4orf50	106479159	45868	RN7SK pseudogene 144	Overlapping
30	rs4570167	EBF2	64641	19090	EBF transcription factor 2	Overlapping
31	rs2024578	RNU4ATAC7P	106480539	46893	RNA, u4atac small nuclear 7, pseudogene	190853
32	rs11050349	TMTC1	83857	24099	Transmembrane O-mannosyltransferase targeting cadherins 1	Overlapping
33	rs4717992	GUSBP6	653435	42320	GUSB pseudogene 6	Overlapping
34	rs4503064	SOX7	83595	18196	SRY-box transcription factor 7	1886
35	rs683466	AL353742.1				71555
36	rs4855447	MITF	4286	7105	Melanocyte inducing transcription factor	Overlapping
37	rs893746	LINC01090	104355152	49201	Long intergenic non-protein coding RNA 1090	Overlapping
38	rs4269571	SLC30A8	169026	20303	Solute carrier family 30 member 8	Overlapping
39	rs62308683	HERC5	51191	24368	HECT and RLD domain containing E3 ubiquitin protein ligase 5	Overlapping
40	rs7112956	FAT3	120114	23112	FAT atypical cadherin 3	Overlapping
41	rs76797216	LINC00700	282980	27422	Long intergenic non-protein coding RNA 700	89426
42	rs34926045	CORO2B	10391	2256	Coronin 2B	142676
43	rs2971760	NDUFB9P2	100128596	52270	NADH:ubiquinone oxidoreductase subunit B9 pseudogene 2	24048
44	exm2260036	NDUFB9P2	100128596	52270	NADH:ubiquinone oxidoreductase subunit B9 pseudogene 2	24048
45	rs7027911	AL592221.1				45961
46	rs2061771	ZNF765	91661	25092	Zinc finger protein 765	Overlapping
47	rs1339374	CCDC107	203260	28465	Coiled-coil domain containing 107	Overlapping
48	rs8009579	ARMH4	145407	19846	Armadillo like helical domain containing 4	Overlapping
49	rs72711389	LINC01098	285501	27731	Long intergenic non-protein coding RNA 1098	3146
50	rs2028964	LINC01098	285501	27731	Long intergenic non-protein coding RNA 1098	3146
51	rs61883849	HSD17B12	51144	18646	Hydroxysteroid 17-beta dehydrogenase 12	Overlapping
52	rs4738067	PRDM14	63978	14001	PR/SET domain 14	Overlapping
53	rs73379947	PTPRM	5797	9675	Protein tyrosine phosphatase receptor type M	Overlapping
54	rs719530	AC092167.1				181331
55	rs4955988	CACNA2D3	55799	15460	Calcium voltage-gated channel auxiliary subunit alpha2delta 3	Overlapping

56	rs78471707	TXNL1P1	100419095	39459	Thioredoxin like 1 pseudogene 1	164399
57	rs2596906	IQSEC1	9922	29112	IQ motif and Sec7 domain arfgef 1	4135
58	rs9309473	ALMS1	7840	428	ALMS1 centrosome and basal body associated protein	Overlapping
59	rs10906653	FRMD4A	55691	25491	FERM domain containing 4A	Overlapping
60	rs58500584	AC097713.2				18741
61	rs10797649	RNVU1-6	101954276	48314	RNA, variant U1 small nuclear 6	12041
62	rs12271908	NCAM1	4684	7656	Neural cell adhesion molecule 1	Overlapping
63	rs6944414	GALNT17	64409	16347	Polypeptide N-acetylgalactosaminyltransferase 17	Overlapping
64	rs67193795	KIF5C	3800	6325	Kinesin family member 5C	Overlapping
65	rs61917776	PKNOX2	63876	16714	PBX/knotted 1 homeobox 2	Overlapping
66	rs78715995	PKNOX2	63876	16714	PBX/knotted 1 homeobox 2	Overlapping
67	rs75412658	CASC15	401237	28245	Cancer susceptibility 15	Overlapping
68	rs76326187	PLEKHG1	57480	20884	Pleckstrin homology and rhogef domain containing G1	2525
69	rs11877530	TNFRSF11A	8792	11908	TNF receptor superfamily member 11a	Overlapping
70	rs73339865	AC106795.3				Overlapping
71	rs742460	GNPTG	84572	23026	N-acetylglucosamine-1-phosphate transferase subunit gamma	Overlapping
72	rs6887695	AC008691.1				Overlapping
73	rs11130310	PBRM1	55193	30064	Polybromo 1	Overlapping
74	rs80225482	PBRM1	55193	30064	Polybromo 1	Overlapping
75	rs4920461	IGSF21	84966	28246	Immunoglobin superfamily member 21	Overlapping
76	rs2153240	SIT1	27240	17710	Signaling threshold regulating transmembrane adaptor 1	1284
77	rs9905906	DNAH2	146754	2948	Dynein axonemal heavy chain 2	Overlapping
78	rs10063787	AC112206.2				Overlapping
79	rs4712460	MBOAT1	154141	21579	Membrane bound O-acyltransferase domain containing 1	Overlapping
80	rs12434181	AL160237.1				78386
81	rs73738318	CLIC5	53405	13517	Chloride intracellular channel 5	Overlapping
82	rs9535171	CDADC1	81602	20299	Cytidine and dcmp deaminase domain containing 1	4420
83	rs3132718	AL645929.2				Overlapping
84	rs61739896	DNAH1	25981	2940	Dynein axonemal heavy chain 1	Overlapping
85	rs4731262	AC003975.1				152380

86	rs28476533	AC068594.1				7975
87	rs12129573	LINC01360	101927295	50593	Long intergenic non-protein coding RNA 1360	3487
88	rs8080678	RPTOR	57521	30287	Regulatory associated protein of MTOR complex 1	Overlapping
89	rs4458115	PTPRM	5797	9675	Protein tyrosine phosphatase receptor type M	Overlapping
90	rs4351004	AC006390.1				93064
91	rs667725	AC000372.1				94623
92	rs682585	RGS16	6004	9997	Regulator of G protein signaling 16	8248
93	rs2997971	RYR2	6262	10484	Ryanodine receptor 2	Overlapping
94	rs6478453	MIR147A	406939	31534	Microrna 147a	211676
95	rs6968910	HECW1	23072	22195	HECT, C2 and WW domain containing E3 ubiquitin protein ligase 1	Overlapping
96	rs32323	RNU6-679P	106479866	47642	RNA, U6 small nuclear 679, pseudogene	165110
97	rs13072556	RF00139				13690
98	rs1932458	SAXO1	158297	28566	Stabilizer of axonemal microtubules 1	Overlapping
99	rs3771003	NRP2	8828	8005	Neuropilin 2	Overlapping
100	rs920590	INTS10	55174	25548	Integrator complex subunit 10	23490
101	rs13224784	AC090114.2				8507
102	rs9999241	SORCS2	57537	16698	Sortilin related VPS10 domain containing receptor 2	19272
103	rs582937	HDAC9	9734	14065	Histone deacetylase 9	Overlapping
104	rs13231717	AC073486.1				23312
105	rs2428918	MAGI2	9863	18957	Membrane associated guanylate kinase, WW and PDZ domain containing 2	643
106	rs11150737	RPTOR	57521	30287	Regulatory associated protein of MTOR complex 1	Overlapping
107	rs77708863	SPOCK1	6695	11251	SPARC (osteonectin), cwcv and kazal like domains proteoglycan 1	Overlapping
108	rs76734228	DLGAP1	9229	2905	DLG associated protein 1	Overlapping
109	rs9297310	NCALD	83988	7655	Neurocalcin delta	Overlapping
110	rs2834623	LINC01426	100506385	50734	Long intergenic non-protein coding RNA 1426	Overlapping
111	rs590616	ARHGAP42	143872	26545	Rho gtpase activating protein 42	Overlapping
112	rs1344542	RN7SKP250	106479206	45974	RN7SK pseudogene 250	18514
113	rs904091	AC093025.1				104120
114	rs77986901	LINC02572	105373615	53634	Long intergenic non-protein coding RNA 2572	316276

115	rs1280414	CGNL1	84952	25931	Cingulin like 1	Overlapping
116	rs77759376	RNF121	55298	21070	Ring finger protein 121	Overlapping
117	rs959193	HNRNPAB	3182	5034	Heterogeneous nuclear ribonucleoprotein A/B	2461
118	rs12778455	RPL34P19	100271494	36925	Ribosomal protein L34 pseudogene 19	6064
119	rs4945848	GPR6	2830	4515	G protein-coupled receptor 6	47654
120	rs61406133	AC079612.1				64383
121	rs60603263	AC131211.1				130564
122	rs13278603	AC090578.1				Overlapping
123	rs6844238	MYOZ2	51778	1330	Myozenin 2	Overlapping
124	rs10922613	GBP6	163351	25395	Guanylate binding protein family member 6	7525
125	rs9737503	B4GALNT4	338707	26315	Beta-1,4-N-acetyl-galactosaminyltransferase 4	33052
126	rs4437352	AC008581.2				Overlapping
127	rs114471390	LINC02572	105373615	53634	Long intergenic non-protein coding RNA 2572	248217
128	rs17657660	CYP2T3P	163007	18853	Cytochrome P450 family 2 subfamily T member 3, pseudogene	1107
129	rs6710692	ALMS1	7840	428	ALMS1 centrosome and basal body associated protein	Overlapping
130	rs651862	SLC24A3	57419	10977	Solute carrier family 24 member 3	Overlapping
131	rs6791903	ZNF385D	79750	26191	Zinc finger protein 385D	Overlapping
132	rs943121	PFKFB3	5209	8874	6-phosphofructo-2-kinase/fructose-2,6-biphosphatase 3	1984
133	rs12721515	CSF1	1435	2432	Colony stimulating factor 1	Overlapping
134	rs115128033	AC237221.2				29828
135	rs4661310	KAZN	23254	29173	Kazrin, periplakin interacting protein	Overlapping
136	rs148373909	DNASE1	1773	2956	Deoxyribonuclease 1	Overlapping
137	rs786201057.1	HSPA8P13	106480803	44928	Heat shock protein family A (Hsp70) member 8 pseudogene 13	424705
138	rs118050771	HHIPL1	84439	19710	HHIP like 1	6751
139	rs76866981	Z82202.2				221521
140	rs77194116	DPYS	1807	3013	Dihydropyrimidinase	Overlapping

Table S3: Literature support for significantly associated T2D genes not listed in T2DKP, DisGeNET and Harmonizome databases

No	Gene Symbol	Gene Function reported in literature	Contribution to T2D
1	CASC15	Associated with diabetic macular edema (Gurung et al, 2022)	Complication
2	CCDC107	Identified as diabetes-related atherogenesis gene (Huang et al, 2020)	Complication
3	CCDC69	Related to PPAR-gamma signaling pathway (Li et al, 2021)	Indirect
4	DNAH2	Associated with type 1 diabetes (Qu et al, 2022)	Indirect
5	GALNT17	Involved in galactose metabolism (Jee et al, 2022)	Indirect
6	GBP6	Marker of insulin secretion and inflammation (Goo et al, 2016)	Direct
7	GSTO1	Regulates insulin biosynthesis in pancreatic beta-cells (Wang et al, 2020)	Direct
8	HERC5	Involved in Gestational diabetes pathophysiology (Tang et al, 2022)	Indirect
9	HHIPL1	Associated with insulin resistance, metabolic syndrome in Mexican Americans (Rogriguez-Perez et al, 2018)	Direct
10	INTS10	Associated with T2D in Arab (Hebbar et al, 2021) and African (Ng et al, 2017) populations	Direct
11	KIF5C	Negatively regulates adipogenesis (Chen et al, 2017)	Indirect
12	MARCH2	May influence serum triglyceride and HDL levels (Sajuthi et al, 2017)	Indirect
13	MIR147A	Alleviates oxidative stress and associated with diabetic nephropathy (Li et al, 2021)	Complication
14	PFKFB3	Involved in diabetic retinopathy (Min et al, 2021)	Complication
15	PRDM14	Significant predictor of new onset diabetes after transplantation (Chand et al, 2016)	Indirect
16	RGS16	Promotes insulin secretion and beta-cell regulation (Vivot et al, 2016)	Direct
17	RNF166	Involved in diabetic retinopathy (Hongbo et al, 2021)	Complication
18	RNU6-679P	Part of splicing machinery associated with T2D progression (Del Rio-Moreno et al, 2020)	Direct
